# Performance of Primary Dental Care Services: An Ecological Study in a Large Brazilian City

**DOI:** 10.1155/2013/176589

**Published:** 2013-12-12

**Authors:** Rita Sibele Souza Esteves, Juliana Vaz Melo Mambrini, Ana Cristina Borges Oliveira, Mauro Henrique Nogueira Guimarães Abreu

**Affiliations:** ^1^Department of Community and Preventive Dentistry, Universidade Federal of Minas Gerais, Belo Horizonte, Avenida Antônio Carlos 6627, 31270901 Belo Horizonte, MG, Brazil; ^2^René Rachou Research Center, FIOCRUZ, Avenida Augusto de Lima 1715, 30190002 Belo Horizonte, MG, Brazil

## Abstract

This study explored the association between area-level primary dental care performance and area-level demographics, dental treatment need, and health care service indicators. An ecological cross-sectional study was performed in Belo Horizonte, Brazil, in 2010. The 142 primary health care (PHC) units were grouped based on the following variables: access to individual dental treatment, frequency of dental emergencies, and frequency of individual preventive procedures. The independent variables analyzed were demographic variables, dental treatment need, and health care service indicators. The data were obtained from the information systems of the Brazilian Ministry of Health and the city of Belo Horizonte. We explored the associations between membership in a specific PHC cluster type and the independent variables using multinomial logistic regression with a significance level of 5%. Variables such as the high/very high vulnerability of population, rate of completed treatment, and rate of referrals of users to secondary care were independently associated with the clusters (*P* < 0.05). The performance of primary dental care services was associated with patient demographics, dental treatment need, and referrals. The results of this study have implications for the planning of public policies.

## 1. Introduction

Health care system performance is an important issue in public health research to support public policy development. The most recent Behavioral Model of Health Services Use stresses that health service use is best understood by focusing on contextual and individual determinants. A range of contextual variables, such as demographic, social, organizational, and population health indices, could be correlated with the process of medical care and use of services [1].

International studies and nationwide studies in Brazil have been conducted to identify the relationships between social and economic factors and the performance of dental services [2–10]. Despite the importance of group-level variables in epidemiological studies [11], most of these studies are conducted on an individual basis. Several ecological studies have been conducted to analyze the relationships among social and economic conditions, the use and access to dental services [2–4, 6, 7], and the prevalence of oral health conditions [6, 7, 12–14]. However, these studies did not analyze intramunicipal differences. There is a lack of ecological studies that assess the factors associated with the performance of primary oral health care in large cities, especially in Brazil. One study, conducted in London, revealed that children and adolescents living in more socially vulnerable neighborhoods had less access to dental services. Access for adults in the same city was directly proportional to the vulnerability of the area [9].

The Brazilian Health System (acronym SUS), a publicly funded health care system, provides universal access and comprehensive care, including oral health care. Although this program has ensured high levels of access to health services for most of the population, the reduction of the social exclusion of specific subgroups remains a challenge to the SUS [15]. To reduce the social inequality in Brazil, health care managers and personnel need to plan their actions based on evaluations of the health status of the population [16] at the local, municipal, state, and federal levels. The assessment of the health system could provide evidence to support the development of new policies designed to reduce social and economic inequalities in health care [17].

Thus, the purpose of this paper is to explore the association between area-level primary dental care performance and area-level demographics, dental treatment need, and health care service indicators.

## 2. Methods 

An ecological cross-sectional study was performed in Belo Horizonte, Brazil, in 2010. The city of Belo Horizonte is the capital of the state of Minas Gerais, located in southeast Brazil. The city has a population of 2,375,151 inhabitants, and fluoridated water has been available in the city since 1975 (0.60 to 0.85 ppm). Similar to many other Brazilian cities, the city has a serious problem with social inequality; the provision of health services represents a strategy for reducing this disparity. The public health system in Belo Horizonte is composed of 146 primary health care units (PHC) designed as preferential entry points for the population to health services. The PHCs offer a range of services, seeking to provide comprehensive care to individuals and the community. The public health system of Belo Horizonte has adopted a family health strategy to serve populations. In 2010, the system included 539 family health teams (FHTs) and 237 oral health teams (OHTs), as well as other services, which were geographically distributed throughout the city [18, 19]. The FHTs and OHTs are responsible for the care of approximately 75.0% of the city's populations. Each OHT provides preventive, restorative, surgical, and emergency dental treatment to the population in the territory.

We used data from the Brazilian national health information system of the Ministry of Health [20] and from the local health system of Belo Horizonte for 142 HC units with OHTs (participation rate = 97.3%) in 2010. The inclusion criterion for each HC was the presence of an OHT. Thus, our study is a census of HCs with OHTs in a large Brazilian city. Data were extracted from health systems by a single researcher with 10 years of experience in these web environments. The data bank was evaluated for inconsistencies by a senior epidemiologist and a biostatistician for data quality control. In cases of inconsistency, the data in the local (city) database were reevaluated and modified.

The formation of clusters (dependent variable) was based on the similarity of variables of access to individual dental care, access to dental emergencies, and the frequencies of specific preventive dental procedures (Table 1).

Five different groupings (with two, three, four, five, or six clusters) were formed from the 142 HCs in the city and compared. The three-cluster model was selected to provide a better understanding of the phenomenon (performance of primary dental care). An agglomerative hierarchy technique based on the Ward method was used for the cluster analysis [21].

The explanatory variables analyzed, based on the Behavioral Model of Health Services Use [1], were demographic factors and epidemiological and health care organization indicators.

Demographic indicators were obtained by calculating the percentage of health service users registered with the city council of Belo Horizonte, Brazil, in the following age ranges [22]: younger than ten years, ten to 19 years, 20 to 59 years, and 60 years or older. The health territories that were classified as very high or high health vulnerability by the Health Vulnerability Index (HVI) were combined and defined as areas of high vulnerability. This indicator was developed to determine the degree of social vulnerability of the population of a determined area within a city and to identify priority areas for intervention and resource allocation, thus contributing to planning with the goal of reducing urban inequality. The HVI was created using sociodemographic data (sanitation, housing, education, income, and age of head of family) and health indicators [20, 21].

Based on the WHO criteria for dental caries [23], trained dentists from the OHTs performed examinations to determine dental treatment need (routinely collected data) and rated the need of each individual on a scale from zero to five. This classification is performed to allow the OHT to prioritize the treatment of users with the greatest need for treatment. On this scale, zero indicates individuals without any cavitated carious lesions; one indicates individuals with one to three teeth with cavitated carious lesions; two through four identify patient with more than three teeth with cavitated carious lesions or who require the extraction of remaining dental elements. Individuals with a score of five have controlled decay but require periodontal treatment. This classification is a modification made by council of Belo Horizonte from a previous study [24]. The overall oral health status of the patients of a given HC was determined by calculating the proportion of individuals classified with each of the codes relative to the total number of individuals examined.

The rate of completed dental treatment was calculated as the percentage of examined patients who had received final treatment for each code (according to dental treatment need) relative to the total number of finalized treatments. The indicator of referrals to secondary oral care was calculated as the percentage of referrals made relative to the number of first dental visits. The population covered by FHTs and by OHTs was calculated by dividing the population of a health territory by the total number of teams [22].

Firstly, bivariate multinomial logistic regression models were developed to identify explanatory variables (age, vulnerability, dental treatment need, rate of complete dental dental treatment, population covered by FHTs and by OHTs, and referrals to secondary care) associated with the dependent variable. Each variable with *P* value lower than 0.20 was included in the multivariate multinomial logistic regression. Explanatory variables with *P* value lower than 0.05 were maintained in the final model. Adjusted odds ratios (CI 95%) were also calculated. Probabilities predicted by the model for each one of the covariates in the final multivariate model were plotted, keeping the others fixed at their median value. The Wald test was used to assess the statistical significance of these relationships. The data were analyzed using Statistical Package for the Social Sciences (SPSS for Windows, version 18.0 SPSS Inc., Chicago, IL, USA) software.

The study was submitted to and approved by the Ethics Committee for Human Research of the Universidade Federal de Minas and the city of Belo Horizonte (protocol no. 0136.0.203.000-11).

## 3. Results

We evaluated 142 HCs with OHTs. No data were missing for any variable in any PHC. The primary dental care performance is described in Table 2. Cluster 1 includes PHCs with the highest average proportion of individual preventive dental procedures. PHCs in cluster 2 were associated with the lowest access to individual dental treatment and the highest average proportion of dental emergencies. PHCs from cluster 3 exhibited the lowest average proportion of preventive dental procedures.

The description of median value of each explanatory variable stratified by clusters is presented in Table 3.

The final multivariate multinomial logistic regression model is shown in Table 4. The increase of one point in the rate of completed treatment (more than 3 teeth with cavities and/or requiring extraction) increased in 6.1% (CI 95% 2.1–10.2) the odds of pertaining to cluster 2 compared to 1. The increase of one point in the rate of completed treatment (more than 3 teeth with cavities and/or requiring extraction) increased in 5% (CI 95% 0.3–10.0) the odds of pertaining to cluster 3 compared to 1.

**Figure 1:**
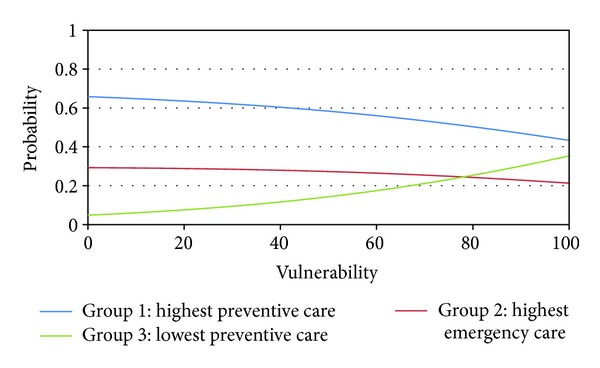
Probability of pertaining to each cluster according to vulnerability in Belo Horizonte, Brazil, 2010.

**Figure 2:**
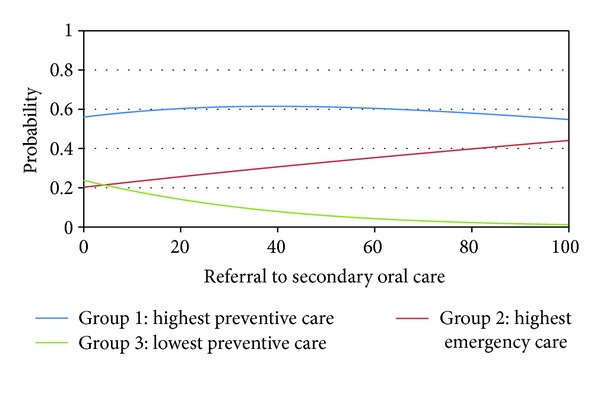
Probability of pertaining to each cluster according to referral to secondary oral care in Belo Horizonte, Brazil, 2010.

**Figure 3:**
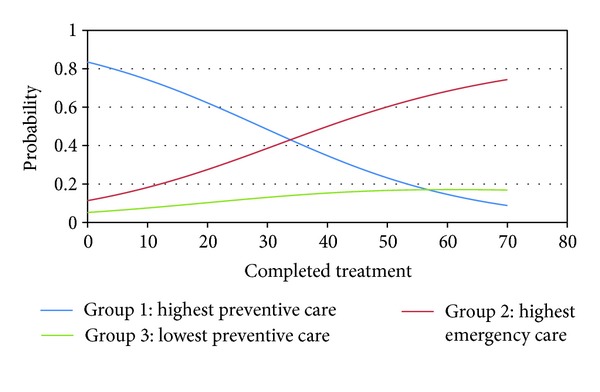
Probability of pertaining to each cluster according to completed treatment (more than 3 teeth with cavities and/or requiring extraction) in Belo Horizonte, Brazil, 2010.

The increase of one point in the rate of vulnerability increased in 2.5% (CI 95% 0.8–4.1) the odds of pertaining to cluster 3 compared to 1. Finally, the increase of one point in the rate of referrals to secondary care increased in 0.971 (CI 95% 0.943–0.999) the odds of pertaining to cluster 3 compared to 1; that is, the decrease of one point in the rate of referrals to secondary care increased in 3% the odds of pertaining to cluster 3 compared to 1. No other explanatory variable was associated with the dependent variable. Figures 1, 2, and 3 showed the association between the probabilities of belonging to each cluster according to the rate of vulnerability, referrals to secondary care, and completed treatment.

## 4. Discussion

We found that in the same city there are different PHCs in terms of performance of primary care services. Some demographic, epidemiological, and health care organization indicators were associated with this performance.

High proportion of high-vulnerability patients in the PHCs of cluster 3 could explain the low proportion of preventive dental procedures. The proportion of individual preventive procedures is calculated as the number of preventive procedures divided by the total of all clinical procedures (preventive, restorative, and surgeries). Therefore, the less the proportion of preventive procedures, the more the proportion of restorative procedures and surgeries. The PHCs that faced greater social vulnerability challenges performed a greater proportion of restorative procedures and surgeries. In general, populations from more vulnerable areas had a greater need for restorative and surgical dental procedures as a result of poor access to preventative dental care [25]. As such, more vulnerable populations may indeed require more of a health team's time to address more immediate needs, such as pain or the restoration of impaired teeth. It is possible that the OHTs in cluster 3 prioritized the treatment of these conditions to the detriment of procedures aimed at disease prevention. The results of the present study inspire a reflection on the equity of dental care. There is a tendency to favor equity in the organization of health services in this large Brazilian city. Additionally, our results suggest that the dental services in the city are aimed at reducing inequality between population groups through the implementation of procedures that are appropriately targeted to the local needs (in this case, restorative and surgical procedures) and through providing services in socially disadvantaged areas. On the other hand, we found that these socially vulnerable populations tend to have fewer resources aimed at the prevention of disease. Considering that these procedures are also important for oral health and oral health quality of life, these results seem to reflect the law of inverse care. The law of inverse care tends to prevail in less equal societies, with better access to health services in populations with more favorable social conditions [26].

The higher rate of completed dental treatment needs identified in the PHCs of cluster 2 and 3 compared to cluster 1could explain their performance of primary dental care services. Services with patients that had greater proportion of decayed and missing teeth are more likely to provide emergency dental services [27, 28] and are less likely to use preventive services. In the same way, high dental treatment needs result in less access to individual dental treatment because the number of dental appointments required for each individual is greater.

In cluster 3, the rate of referrals to secondary oral care is inferior to cluster 1, independent of other explanatory variables. In cluster 3 there was higher rate of completed dental treatment needs (more than 3 teeth with cavities and/or requiring extraction) and higher social vulnerability. It is necessary to investigate the organizational, epidemiological, and/or social characteristics of these PHCs in order to identify the difficulties of referring patients to secondary care. Qualitative research could be useful to investigate this issue.

Other variables in the Behavior Model of Health Services Use were tested. However, no further variables were associated with the performance of primary dental care services. The clusters were homogenous in terms of age group distribution. The population coverage per family health team and per oral health team could in theory affect the performance of the services. However, this indicator had no influence on the primary dental care performance.

In this city, the dental care performance was closely associated with the HVI of the area. The growth of health resources (physical, human, technological, and equipment) in more vulnerable areas may contribute to the implementation of preventive procedures, which will reduce the prevalence of oral disease in the long term.

This study has some limitations that should be addressed. The cross-sectional methodology does not permit the inference of causal relationships. Data for each variable were collected by different public health care providers and reproducibility cannot be accessed. We do not evaluate private dental practice. However, because ecologic studies allow the analysis of the impact of contextual variables, this approach can be of great use, considering the need to evaluate the effects of health policies [29].

Our results also indicate that goals for oral health, even within the same city, should be different. Global oral health goals, objectives, and targets from the WHO indicate the need for a framework at different levels—regional, national, and local [30].

The evaluation of indicators of access to service and the monitoring of oral disease may enhance health interventions by directing additional resources to the areas of greatest need. Ecological studies that evaluate health services in a large, socially diverse city can contribute to the understanding of the effects of inequalities in health care. These studies facilitate the planning of health care services and an improved distribution and allocation of resources in this and other health care systems worldwide.

## 5. Conclusions 

The performance of primary dental care services was associated with patient demographics, dental treatment need, and referrals. The results of this study have implications for the planning of more inclusive public policies.

## Figures and Tables

**Table 1:** Indicators used for cluster formation, Belo Horizonte, 2010.

Indicators	Description
Access to individual dental care	Proportion of residents who receive a scheduled primary dental consultation with the aim of diagnosing and elaborating a preventive/therapeutic plan to address the detected needs, calculated as a percentage of the population registered by the Belo Horizonte Social Census.
Frequency of dental emergencies	The indicator was obtained by calculating the percentage of dental emergency consultations in primary health care (i.e., pulpal pain, acute periapical abscess, pericoronitis, periodontal abscess, dental trauma, etc.) relative to the total number of dental consultations in primary health care.
Proportion of individual preventive procedures	The indicator was obtained by calculating the percentage of preventive dental procedures (plaque control, fluoride treatment, sealants, dental scaling, patient counseling, and supervised individual brushing) relative to the total number of dental procedures in primary health care (preventive, restorative, and surgical procedures).

**Table 2:** Description of primary dental care performance in each cluster, Belo Horizonte, 2010.

Cluster	Number of PHCs	Average access to individual dental treatment (minimum–maximum)	Average proportion of dental emergencies (minimum–maximum)	Average proportion of preventive individual procedures (minimum–maximum)
1 (highest preventive care)	78	7.1% (1.8%–24.3%)	9.5% (1.7%–15.8%)	66.7% (53.7%–81.6%)
2 (highest emergency care)	43	5.7% (1.0%–12.6%)	24.2% (16.9%–41.4%)	62.8% (44.9%–81.6%)
3 (lowest preventive care)	21	7.1% (0.7%–13.4%)	15.8% (3.4%–37.8%)	43.4% (28.7%–52.1%)

**Table 3:** Median values of demographic and dental treatment need indicators and health care services indicators in each cluster, Belo Horizonte, 2010.

Explanatory variables (median values)	Cluster 1	Cluster 2	Cluster 3
High/very high vulnerability	31.5	32.3	74.6
<10 years	9.5	9.0	10.2
10 to 19 years	15.7	16.2	17.1
20 to 59 years	60.5	60.3	59.9
60 years and above	14.2	14.1	11.2
No teeth with cavities	16.8	12.5	17.4
Up to 3 teeth with cavities	43.5	41.9	46.2
More than 3 teeth with cavities and/or requiring extraction	28.6	33.6	34.5
Necessity of periodontal treatment	7.2	5.9	3.5
Completed treatment (no teeth with cavities)	24.1	21.5	27.3
Completed treatment (up to 3 teeth with cavities)	46.9	43.7	45.1
Completed treatment (more than 3 teeth with cavities and/or requiring extraction)	18.5	24.4	27.0
Completed treatment (periodontal treatment)	5.8	4.8	3.5
Referral to secondary oral care	32.5	33.7	16.0
Population coverage per family health team	2930.3	2936.8	2855.7
Population coverage per oral health team	8075.0	8972.5	6813.5

**Table 4:** Multivariate multinomial logistic regression, Belo Horizonte, 2010.

Variables	Cluster 2 × Cluster 1		Cluster 3 × Cluster 1	
	OR (95% CI)	*P* value	OR (95% CI)	*P* value
Vulnerability	1.001 (0.988–1.014)	0.872	1.025 (1.008–1.041)	0.003
Referrals to secondary care	1.008 (0.992–1.023)	0.333	0.971 (0.943–0.999)	0.045
Completed treatment (more than 3 teeth with cavities and/or requiring extraction)	1.061 (1.021–1.102)	0.003	1.050 (1.003–1.100)	0.039
